# Cutaneous dysbiosis may amplify barrier dysfunction in patients with atopic dermatitis

**DOI:** 10.3389/fmicb.2022.944365

**Published:** 2022-11-14

**Authors:** Margaret Hammond, Ahmed Gamal, Pranab K. Mukherjee, Giovanni Damiani, Thomas S. McCormick, Mahmoud A. Ghannoum, Susan Nedorost

**Affiliations:** ^1^Department of Dermatology, University Hospitals Cleveland Medical Center/Case Western Reserve University School of Medicine, Cleveland, OH, United States; ^2^Department of Biomedical, Surgical and Dental Sciences University of Milan, Milan, Italy; ^3^Clinical Dermatology, IRCCS Istituto Ortopedico Galeazzi, Milan, Italy; ^4^Department of Pharmaceutical and Pharmacological Sciences, PhD Degree Program in Pharmacological Sciences, University of Padua, Padua, Italy

**Keywords:** atopic dermatitis, microbiome, cutaneous dysbiosis, *Alternaria alternata*, biofilm

## Abstract

Atopic dermatitis (AD) is associated with cutaneous dysbiosis, barrier defects, and immune dysregulation, but the interplay between these factors needs further study. Early-onset barrier dysfunction may facilitate an innate immune response to commensal organisms and, consequently, the development of allergic sensitization. We aimed to compare the cutaneous microbiome in patients with active dermatitis with and without a history of childhood flexural dermatitis (atopic dermatitis). Next-gen Ion-Torrent deep-sequencing identified AD-associated changes in the skin bacterial microbiome (“bacteriome”) and fungal microbiome (“mycobiome”) of affected skin in swabs from areas of skin affected by dermatitis. Data were analyzed for diversity, abundance, and inter-kingdom correlations. Microbial interactions were assessed in biofilms using metabolic activity (XTT) assay and scanning electron microscopy (SEM), while host-pathogen interactions were determined in cultured primary keratinocytes exposed to biofilms. Increased richness and abundance of *Staphylococcus, Lactococcus*, and *Alternaria* were found in atopics. *Staphylococcus* and *Alternaria* formed robust mixed-species biofilms (based on XTT and SEM) that were resistant to antifungals/antimicrobials. Furthermore, their biofilm supernatant was capable of influencing keratinocytes biology (pro-inflammatory cytokines and structural proteins), suggesting an additive effect on AD-associated host response. In conclusion, microbial inter-kingdom and host-microbiome interactions may play a critical role in the modulation of atopic dermatitis to a greater extent than in non-atopic adults with allergic contact dermatitis.

## Introduction

Atopic dermatitis (AD) is a chronic condition of the skin with periodic cutaneous flares frequently accompanied by respiratory symptoms (atopic march) such as allergic asthma or rhinitis (Thestrup-Pedersen, 2000). AD is a multifactorial disease, in which changes in microbial flora, skin barrier defects, and dysregulation of innate and adaptive immunity have been noted (Jinnestal et al., 2014; Nedorost, 2020). Culture-based studies have previously associated AD flares with distinct populations of cutaneous bacteria (e.g., *Staphylococcus aureus*) and fungi (e.g., *Malassezia* and *Candida*) (Ramirez De Knott et al., 2005, 2006; Kong et al., 2012; Chandra et al., 2016) which seem to play a potentially prominent role in cutaneous sensitization (Ramirez De Knott et al., 2005, 2006).

Cutaneous contact sensitization led to durable antigen-specific Th2 responses in humans with AD (Newell et al., 2013), suggesting that the “atopic march” originates as a response to allergic contact dermatitis. In the context of this predisposition to cutaneous sensitization, commensal organisms may colonize and sensitize patients with AD (Ramirez De Knott et al., 2006). The progression of atopic dermatitis is strongly linked to defects in skin barrier function, reduced levels of antimicrobial peptides (AMPs), and an increased local inflammatory response (Werfel et al., 2016). Production of AMPs can be positively regulated by TNF-α and IL-1β, and negatively regulated by TSLP (Lee et al., 2016; Li et al., 2016).

Sequencing analyses allow the characterization of skin microbiota and have largely focused on the bacterial microbiota (bacteriome) (Kong et al., 2012; Seite et al., 2014). Although bacteria coexist with fungi in most body sites colonized by microbes, the role of the fungal microbiome (mycobiome) in AD has been investigated less thoroughly.

Previous studies have compared the bacteriome and mycobiome in healthy patients to those with AD, which we define as a history of childhood-onset flexural dermatitis. Genetically induced barrier dysfunction and the resultant irritant dermatitis generate chronic innate immune signals leading to sensitization to weakly potent allergens including proteins with Th2 skewing (Kohli and Nedorost, 2016; Nedorost, 2020). Our current study is the first to characterize the bacteriome and mycobiome profiles of affected and unaffected skin in patients with a history of childhood-onset flexural dermatitis (atopic dermatitis) compared with those without childhood flexural dermatitis but with active dermatitis, under investigation for allergic contact dermatitis (non-atopic patients). Comparison of two cohorts with dermatitis but with a different history of the time of onset helps us to understand the factors of type and duration of innate immunity and relationship with communities of cutaneous commensal organisms.

This is important because dermatitis is increasingly recognized as a genetic (e.g., AD) and environmental (e.g., allergic contact dermatitis) disease with considerable overlap between the groups. We hypothesized that patients with childhood-onset flexural dermatitis (aka “atopic dermatitis”) were more likely to be sensitized by organisms in the cutaneous microbiome that impair the already dysfunctional cutaneous barrier. The implication of this is that patients sensitized to the cutaneous microbiome are less likely to benefit from avoidable allergens in the workplace or in personal care products, more likely to benefit from immunomodulatory treatments such as monoclonal antibodies or JAK-STAT inhibitors, as well as from topical or systemic treatments that aim to improve the diversity of the microbiome, decrease pathogen concentration, and break or dissolve biofilm.

We conducted a proof-of-concept pilot study to characterize the skin bacteriome and mycobiome of atopic vs. non-atopic patients with active dermatitis. We also identified inter-kingdom correlations, determined whether bacteria and fungi interact as biofilms, and investigated whether these biofilms secrete biomolecules that influence the production of cytokines and structural proteins in primary keratinocytes.

## Results

### Clustering and diversity analyses

**Figure 1:**
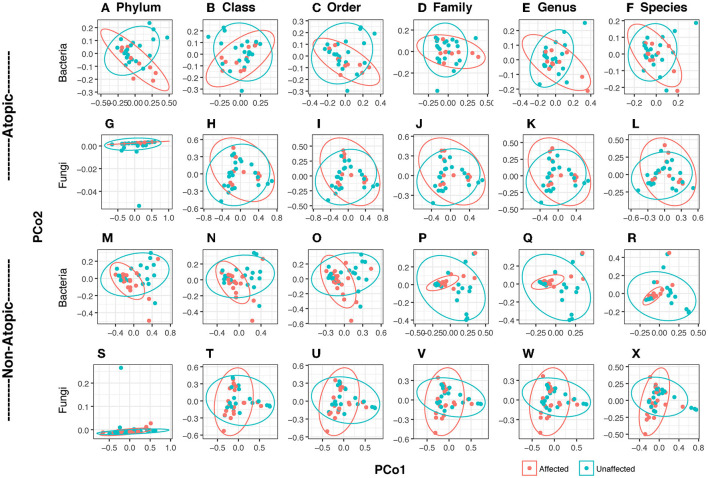
Principal coordinates analysis (PCoA) of bacteriome and mycobiome in skin swabs of atopic and non-atopic patients. Clustering at all taxa levels (Phylum, Class, Order, Family, Genus, and Species) is shown. Ellipses are drawn at a confidence level of 0.95. **(A–L)** Atopic, **(M–X)** non-atopic, **(A–F,M–R)** bacteria, **(G–L,S–X)** fungi. Principal coordinates analysis (PCoA) was conducted with identified reads/OTUs using classical multidimensional scaling (functions *dist*/Euclidean, *cmdscale*) to analyze the distribution of dissimilarities and hierarchical clustering in dendrograms in *vegan* (Oksanen et al., 2017). Homogeneity of groups and beta diversity was evaluated using *adonis* (which explores the differences in group means and implements a multivariate analysis of variances using distance matrices) and *betadisper* (which studies the differences in group homogeneities, similar to Levene's test of the equality of variances) functions in *vegan*.

Bacterial and fungal DNA sequencing produced 1.59 and 1.77 million raw sequences, respectively. In Atopics, skin bacteriome formed clusters which exhibited partial overlap between affected and unaffected sites (Figures 1A–F), while the mycobiome exhibited complete overlap (Figures 1G–J). Among the non-atopics, this pattern in clustering was reversed, with bacteria showing complete overlap (Figures 1M–R), while the mycobiome exhibited clustering with partial overlap (Figures 1S–X). However, there was no significant difference in group homogeneities (based on the distance to the centroid) between affected and unaffected skin in both AD and non-atopic skin (*P* ≥ 0.11). Three principal components accounted for variability in the bacteriome at the phylum level, while in the mycobiome, 6 and 13 fungal species accounted for 99% variability in affected and unaffected samples, respectively.

The richness of affected skin was significantly lower than that of unaffected skin at the Class level (*P* = 0.03) and a trend at the phylum level (*P* = 0.054). Shannon diversity index (SDI) among patients with AD was similar between the affected and unaffected areas. There were no significant differences in diversity between affected and unaffected areas in non-atopics (Supplementary Figure 1).

### Core bacteriome and mycobiome

**Figure 2:**
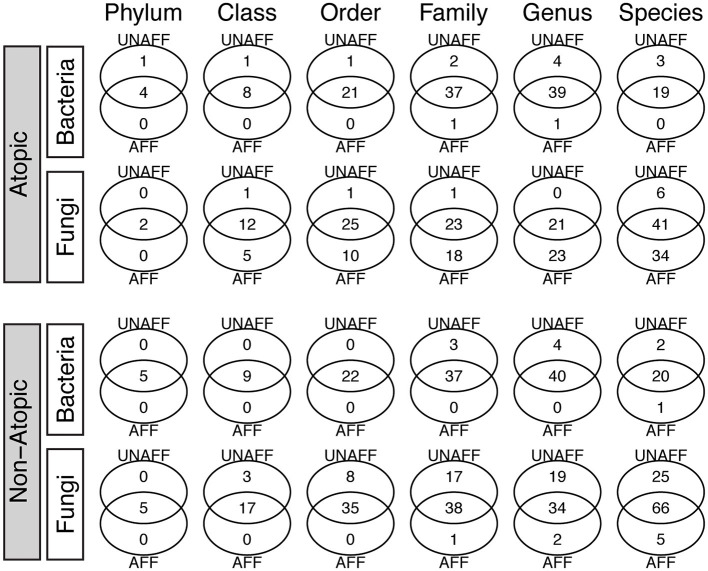
Core bacteriome and mycobiome in affected (AFF) and unaffected (UNAFF) samples of atopic and non-atopic patients. Numbers indicate the actual count of members identified in each taxon (Phylum through Species). The core biome comprised taxa with a frequency of at least 20% (and relative abundance ≥1%).

The core bacteriome (comprising taxa detected with a frequency of at least 20%, at an abundance >1%) comprised four unique Genera (*Gemella, Veillonella, Fusobacterium, Leptotrichia*) in unaffected samples and one (*Leuconostoc*) in affected AD samples, while the core mycobiome comprised two and five phyla in AD and non-atopic subjects, respectively (Figure 2). Interestingly, affected AD samples contained higher numbers of unique fungal genera (23), compared to unaffected samples (0), while in non-atopic samples, unaffected samples had a larger number of unique genera (19) compared to affected samples (2). Similar patterns were observed for other taxa levels (Figure 2).

### Abundance profile of bacteriome and mycobiome

**Figure 3:**
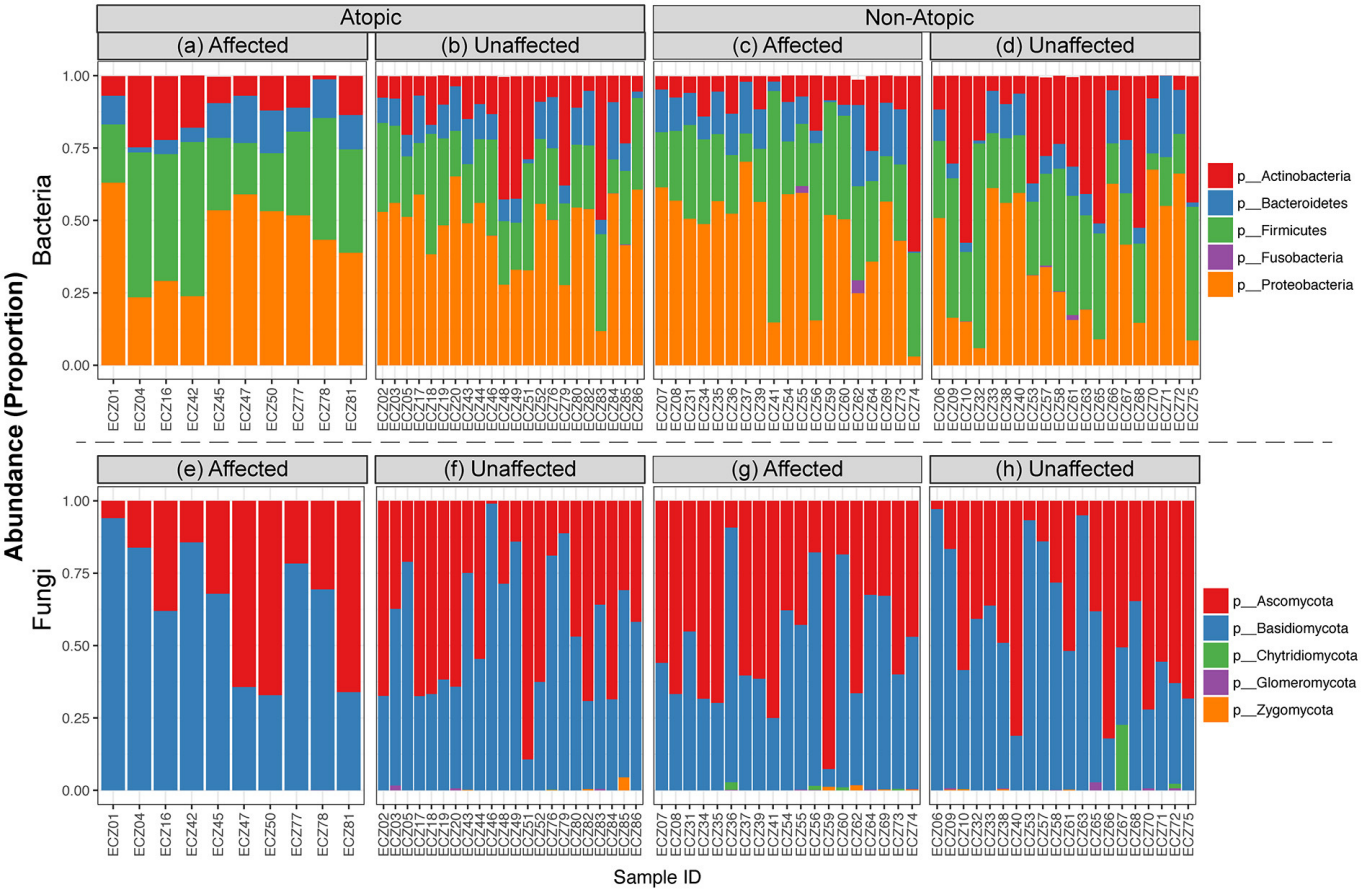
Relative abundance of bacterial and fungal taxa in affected and unaffected skin in atopic and non-atopic dermatitis. Stacked bar charts showing the distribution of **(a–d)** bacterial and **(e–h)** fungal phyla across affected and unaffected samples collected from atopic and non-atopic patients.

Analysis of the abundance distribution of different bacterial and fungal phyla in AD and non-atopic subjects revealed three bacterial (Actinobacteria, Firmicutes, and Proteobacteria) and two fungal (Ascomycota and Basidiomycota) phyla dominating the bacteriome and mycobiome, respectively (Figure 3).

**Figure 4:**
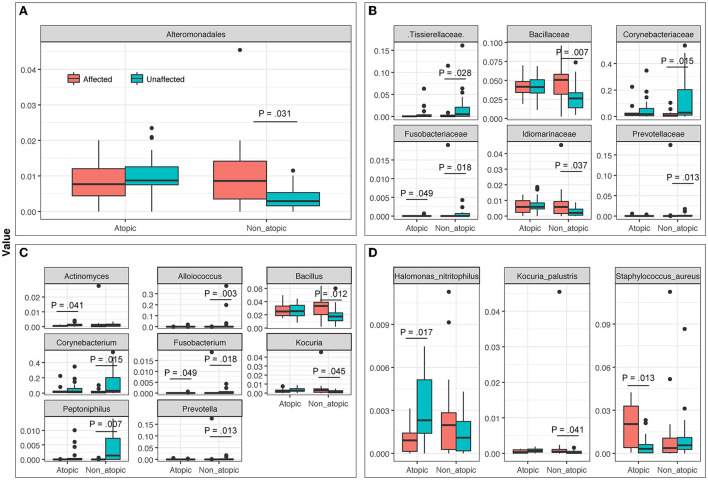
The abundance of bacterial taxa at **(A)** Order, **(B)** Family, **(C)** Genus, and **(D)** Species levels in affected and unaffected skin of atopic and non-atopic patients. Taxa that exhibited statistically significant differences (*P* < 0.05) are shown.

Among samples from AD subjects, abundance levels of bacteria were significantly different at Family, Genus, and Species levels between affected and unaffected skin in both AD and non-atopic subject samples (*P* ≤ 0.049 and =0.013, respectively) (Figure 4).

Furthermore, several known allergenic or pathogenic fungal genera showed noticeable trends in patients with AD, where levels of *Alternaria, Scleroderma*, and *Fusarium* were increased in the affected skin, compared to unaffected skin (Supplementary Figure 2).

### Genus-level abundance comparison

**Figure 5:**
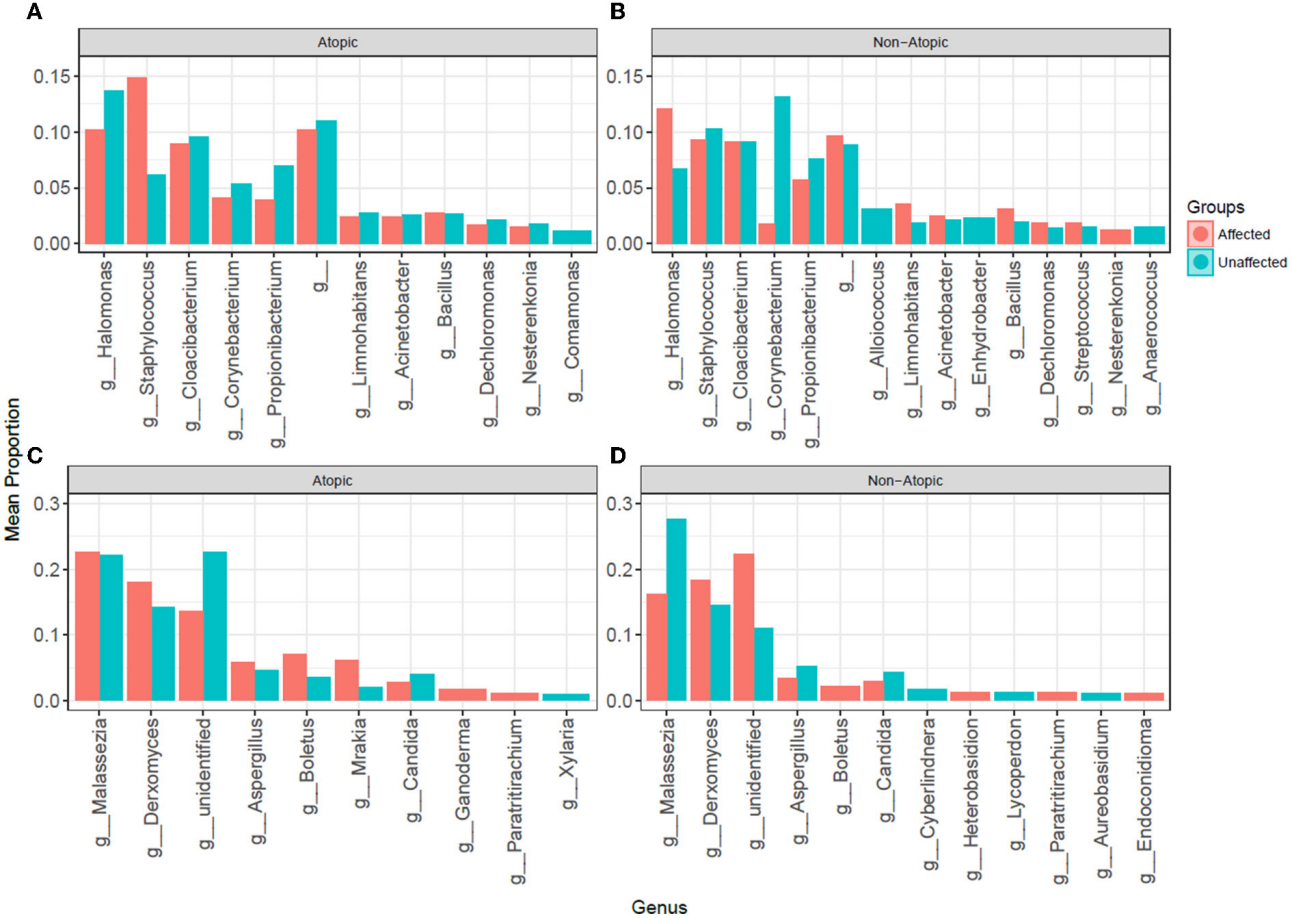
The abundance of **(A,B)** bacterial and **(C,D)** fungal genera in affected and unaffected skin of patients with atopic and non-atopic dermatitis.

The most abundant bacterial genera in Atopics were *Staphylococcus* and *Halomonas* in affected and unaffected skin, respectively (Figure 5). In Non-Atopic patients, *Halomonas* and *Corynebacterium* were the most abundant bacterial genera in affected and unaffected skin, respectively (Figure 5). A comparison of mean proportions revealed that 24 and 27 genera were significantly different in the Atopic and Non-Atopic groups, respectively. These genera included *Staphylococcus* and *Lactococcus*, which were detected at significantly elevated levels in affected skin (*P* = 0.030 and 0.025, respectively), while the abundance of *Bacteroides* was significantly decreased in affected skin (*P* = 0.049), compared to unaffected skin in patients with Atopic dermatitis (Supplementary Table 3). In contrast, in the patients with Non-Atopic dermatitis, an abundance of *Bacillus* and *Brevibacillus* was significantly increased while that of *Corynebacterium* was significantly decreased in affected vs. unaffected skin (*P* ≤ 0.018, Table 1). The abundance of *Fusobacterium* was significantly decreased in affected skin in both atopic and non-atopic groups, compared to unaffected skin (*P* = 0.049 and 0.018, respectively).

**Figure 6:**
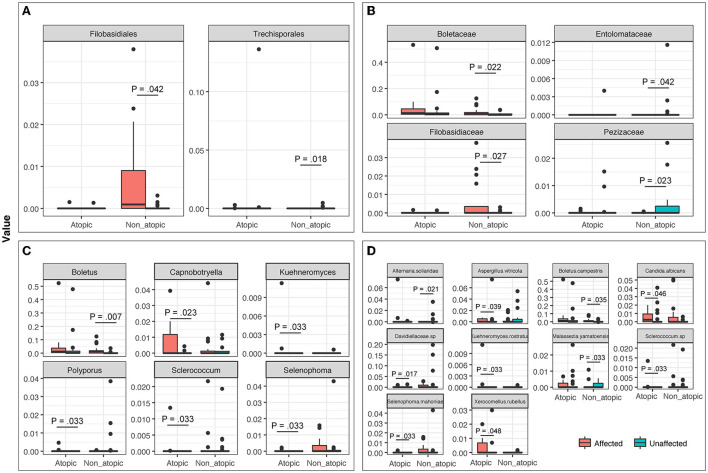
The abundance of fungal taxa at **(A)** Order, **(B)** Family, **(C)** Genus, and **(D)** Species levels in affected and unaffected skin of atopic and non-atopic patients. Taxa that exhibited statistically significant differences (*P* < 0.05) are shown.

The mycobiome also exhibited specific significant differences between affected and unaffected skin in AD and non-atopic subject samples (*P* ≤ 0.048). A statistically significant increase in the abundance of *Candida albicans* was observed in affected samples from AD skin (*P* = 0.046), which was also seen in non-atopic samples, however, the abundance in non-atopic samples did not reach significance (Figure 6).

Mycobiome analysis also revealed that *Malassezia* was the most abundant fungal genus in the affected and unaffected skin of atopics and the unaffected skin of non-atopics (Figure 4). There was no significant difference in the abundance of fungal genera between affected and unaffected skin in both patient groups. However, several known allergenic or pathogenic fungal genera showed a noticeable trend in Atopics, where levels of *Alternaria, Scleroderma*, and *Fusarium* tended to increase in the affected skin, while that of *Trichosporon* tended to decrease, compared to unaffected skin (Supplementary Figure 2).

### Fold changes in genus abundance between affected and unaffected skin

Next, we determined the fold change in the abundance of bacterial and fungal genera between affected and unaffected skin in the two subject groups.

*Staphylococcus* abundance was increased by 2.4-fold in affected skin compared to unaffected areas in patients with AD (14.8 vs. 6.1%, respectively, *P* = 0.030), but not in non-atopic controls (9.3 vs. 10.3%, respectively).

In the mycobiome, the highest fold change was observed for *Alternaria*, which was elevated by 137-fold in affected skin compared to unaffected skin of patients with AD (0.97 vs. 0.007%, respectively, *P* = 0.18), but decreased by 15-fold in non-atopic subjects (0.03 vs. 0.45%) (Supplementary Figure 2).

### *Staphylococcus* and *Alternaria* form robust, drug-resistant, mixed-species biofilms

Our results showed that in patients with AD, *Staphylococcus* abundance was significantly elevated in affected skin. Additionally, the fold change in abundance between affected and unaffected AD skin was the highest for *Alternaria*, an allergenic fungus previously associated with asthma, a key manifestation of adult AD and the “atopic march” (Knutsen et al., 2012; Celakovska et al., 2015; Fukutomi and Taniguchi, 2015). Allergic sensitization through the skin is facilitated by a high concentration of antigen per unit area of inflamed skin. Therefore, we determined whether these two genera interact with each other to form biofilms. *Alternaria* formed robust biofilms as determined by scanning electron microscopy (SEM) and metabolic activity assay. SEM analyses showed that *Alternaria alternata* and *Staphylococcus aureus* were able to form robust biofilms when grown singly (Supplementary Figures 3A,B). However, in the presence of *Staphylococcus, Alternaria* biofilms were increased (Supplementary Figures 3C–E). Notably, mixed biofilms comprised bacterial cells closely aggregating on the fungal hyphae and distinctly enmeshed into the extracellular matrix (Supplementary Figures 3C,D). Fungal biofilm formation increased in the presence of bacterial cells, in a dose-dependent manner (Supplementary Figure 3E). A significant increase in biofilm formation was observed in the presence of 10^3^ and 10^5^ *S. aureus* cells, compared to *Alternata* biofilms formed in the absence of *S. aureus* (*P* ≤ 0.00015, for biofilms formed in presence of both bacterial densities, compared to biofilms formed with no bacteria).

### Biofilms constituents that influence cytokine production in keratinocytes

**Figure 7:**
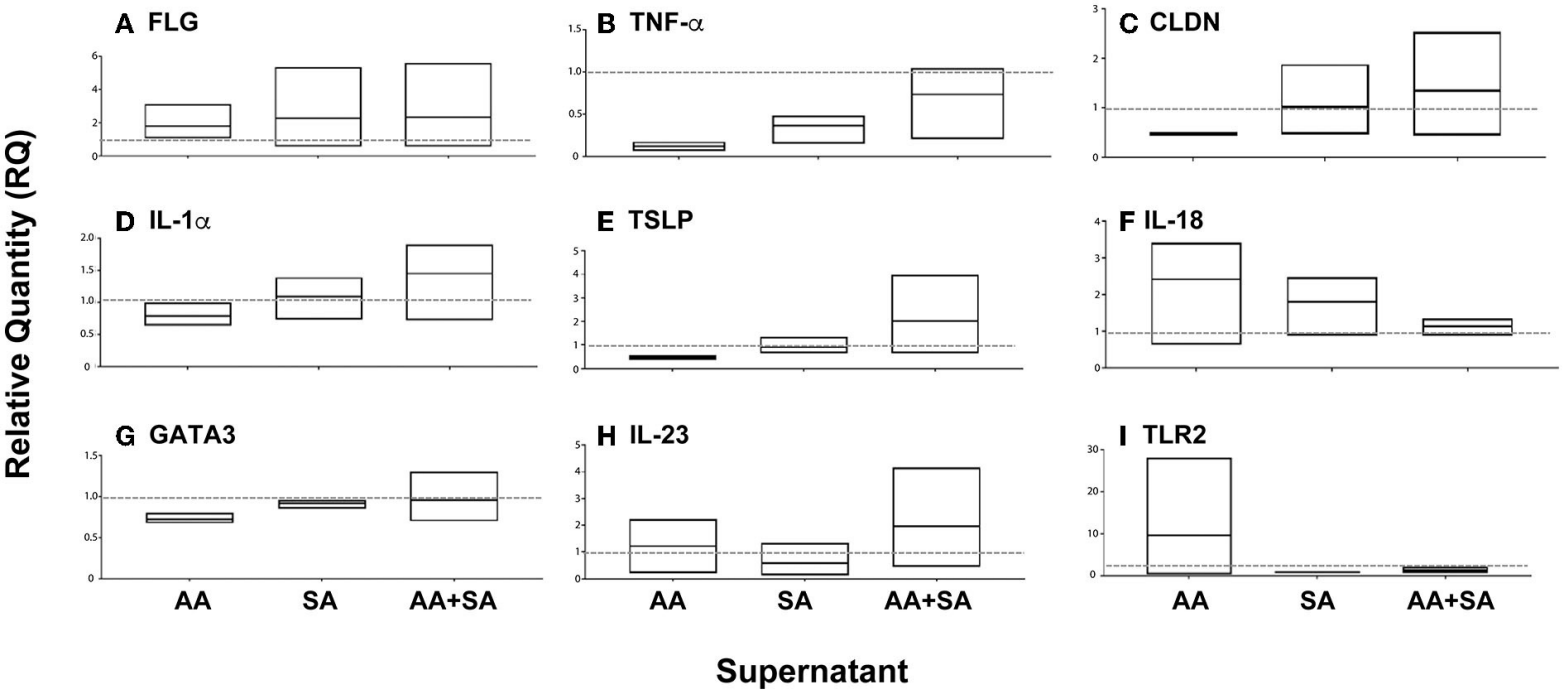
Effect of supernatants of single or mixed-species biofilms on the expression of cytokines and structural proteins in cultured keratinocytes. Biofilms were formed with *Alternaria alternata* (AA), *Staphylococcus aureus* (SA), or both (AA+SA), and their supernatants were added to cultured keratinocytes. Real-time PCR was used to evaluate the expression of specific cytokines and structural proteins. Average fold change (Min to Max, *n* = 3) with the mean represented in the floating boxes are shown compared to the normalized value for controls (dashed line at Fold Change 1).

Next, we determined whether mixed-species biofilms formed by *S. aureus* and *A. alternata* secrete factors that influence the expression of host variables associated with AD including thymic stromal lymphopoietin (TSLP) (known to be highly expressed in the skin of patients with AD and believed to activate downstream immune signals) (Komine, 2009) and pro-inflammatory cytokines (IL-18, IL-23, TNF-α) in keratinocytes (Werfel et al., 2016) (Figure 7).

Supernatants isolated from biofilms formed by *A. alternata* induced a nearly ten-fold increase in mRNA expression of Toll-Like Receptor 2 (TLR2) levels (pattern recognition receptors that are involved in the immune response against different pathogenic microorganisms), with minimal increase in IL-23 and IL-18 mRNA expression levels. Furthermore, exposure to this supernatant also reduced the expression levels of Claudin-1 (CLDN1), which encodes the claudin responsible for the paracellular barrier at tight junctions in the epidermis, and its reduction was reported to play a major role in AD (Tokumasu et al., 2016). Additionally, a reduction in GATA-binding protein 3 (GATA3) (transcription factor for Th2 differentiation and expression of IL-4, IL-13, and IL-5 by T lymphocytes) was also noted.

On the other hand, supernatants from *S. aureus* biofilms reduced the expression of TLR2, IL-18, and IL-23.

Interestingly, supernatant from mixed species (AA + SA) biofilms increased the expression of TSLP, CLDN1, and IL1α, while reducing the expression of TLR2.

These results suggested an additive effect of *A. alternata* and *S. aureus* on the production of AD-associated cytokines and structural proteins by keratinocytes.

## Discussion

Studies over the past two decades have shown that alteration in the skin microbiome is one of the contributors to both AD pathogenesis (initializing and maintenance), AD flares, and AD severity (Faergemann, 2002; Sugita et al., 2010). Analysis of the skin microbiome in patients with AD demonstrated reduced diversity of the bacterial community and an increase in the abundance of *S. aureus* in the affected skin (Kong et al., 2012; Seite et al., 2014). Interestingly, this alteration was also reported to be associated with increasing disease severity, especially during disease flares (Kong et al., 2012). Unfortunately, most studies ignore the fungal community inhabiting our skin, although differences have been reported between patients with skin disorders and healthy individuals (Zhang et al., 2011; Jo et al., 2017; Han et al., 2018). Recent studies have shown evidence of the interaction between bacterial and fungal communities and how they can affect each other in health and disease (Peleg et al., 2010; Diaz et al., 2014; Deveau et al., 2018; Kruger et al., 2019). Thus, studies should not focus solely on bacterial populations but should also include the fungal community, known as the mycobiome.

Our data showed that the abundance of *Staphylococcus* was significantly increased in affected compared to unaffected skin, similar to the observations of other studies (Leyden et al., 1974; Aly et al., 1977; Bibel et al., 1977; Gloor et al., 1982; Higaki et al., 1999). This observation was in agreement with a meta-analysis, that included 81 studies, conducted by Totte et al. (2016) investigating the prevalence of *S. aureus* skin colonization in patients with AD. Furthermore, these data may explain the higher risk of AD-affected skin being impetiginized by *S. aureus*. Seventy percent of the patients included in the analysis carried *S. aureus* on the affected skin and 39% on the unaffected skin (Totte et al., 2016). In healthy individuals, *S. aureus* skin colonization was reported less frequently (Goh et al., 1997). *S. aureus* has been hypothesized to function as a superantigen in AD (Travers et al., 2010; Brauweiler et al., 2013, 2014; Giai et al., 2013; Nakamura et al., 2013; Travers, 2014; Williams and Gallo, 2015). *S. aureus* superantigens bind to major histocompatibility complex class II on keratinocytes causing increased production of pro-inflammatory cytokines including TNF-α and IL-1β secondary to non-specific activation of up to 20% of naïve T cells in the skin (Kim et al., 2006).

We analyzed the mycobiome composition in patients with AD compared to patients with adult-onset allergic contact dermatitis. Interestingly, the fungal genus *Alternaria*, a known fungus that has been linked to different allergic disorders including AD and asthma, exhibited the highest fold-increase in affected skin compared to unaffected skin exclusively in patients with a history of childhood-onset flexural dermatitis (patients with AD). *Alternaria* has also been associated with AD in other studies (Hedayati et al., 2009; Zhang et al., 2011; Han et al., 2018). Moreover, 16 *A. alternata* allergens have been linked to allergenic asthma and increased severity of asthma symptoms (Kustrzeba-Wojcicka et al., 2014; Sharpe et al., 2015; Gabriel et al., 2016).

Thus, we hypothesized that some fungal antigens may play a role in skin sensitization and subsequently share in the development of allergy. This concept is supported by the data provided by Strid et al. (2005) which showed that epicutaneous exposure of mice to peanut protein resulted in a potent Th2-type immunity. Moreover, high levels of IL-4 and serum IgE were also detected. Interestingly, an oral challenge with the same proteins induced a further stronger immune response.

In a study by Walker et al. (2018), *Alternaria* extracts were used for the induction of food allergy in neonatal mice heterozygous for the filaggrin mutation. Mice challenged with food allergens following skin sensitization with *Alternaria* extracts suffered from anaphylaxis compared to controls. Furthermore, skin barrier mutation was necessary for the induction of food allergy in mice's offspring. Filaggrin deficiency and sensitization with *Alternaria* enhance further sensitization to food, and potentially, to aeroallergens.

Buelow et al. (2021) have identified two pathways by which *Alternaria* can cause food allergic reactions using the same mouse model; an IL-33 pathway and a pathway involving oncostatin M (OSM) and amphiregulin (Areg), all of which have been reported to play a role in AD (Peng et al., 2018; Walker et al., 2018; Hashimoto et al., 2019; Scheffschick et al., 2019; Rojahn et al., 2020; Buelow et al., 2021). In the Buelow study, exposure to Alternaria increased the expression of IL-33, OSM, and Areg genes. In addition, these genes were expressed by some keratinocytes which were found in larger numbers in mice with mutations affecting the skin barrier compared to wild-type mice. In addition, the three genes were expressed by a variety of cells including cluster 6 keratinocytes which were found in larger numbers in mice with mutated skin barriers compared to the WT (Buelow et al., 2021). Moreover, increased levels of Areg in keratin-deficient keratinocytes were found to induce the production of TSLP through epidermal growth factor receptor signaling (Scheffschick et al., 2019).

Our data also showed that the supernatant from *Alternaria* or *S. aureus* biofilms alone slightly increased the TSLP message levels. On the other hand, supernatant from mixed species biofilms significantly increased the TSLP expression. This may suggest a synergistic interaction between the two species which supports our electron microscope and XTT assay observations.

Moreover, *Alternaria* biofilm supernatant was found to reduce expression of CLDN1 (a major component of epidermal tight junctions), which has been reported to be significantly reduced in affected AD skin compared to healthy controls, together with significant decreases in tight junction barrier of the skin in a dose-dependent manner (Bergmann et al., 2020). Additionally, in a CLDN1 knock-down (KD) *in vitro* 3D model of a reconstructed human epidermis that expressed different levels of CLDN1, intercellular lipid lamellae length was significantly reduced after CLDN1 KD at day 8, with a significant downregulation of filaggrin mRNA (a protein produced by keratinocytes which have been linked to several allergic diseases including AD) (Nomura et al., 2008; Thyssen and Kezic, 2014; Bergmann et al., 2020). Interestingly, downregulation of CLDN1 has also been reported to induce the production of pro-inflammatory cytokines as well as promote the inflammatory effects of non-pathogenic *Staphylococci* (Tokumasu et al., 2016; Bergmann et al., 2020). Despite our small cohort of patients, the observed trends mark points of departure for further exploration of the role of inter-kingdom interactions in the pathophysiology of AD.

The existence of inter-kingdom interactions between *Alternaria* and *Staphylococcus* was demonstrated by biofilm studies (Figure 7). The presence of biofilms likely explains the difficulty in eradicating *S. aureus* in patients with AD. The presence of bacteria-fungi mixed species biofilms may provide a protective niche to both, thereby affecting skin function and resulting in skin barrier disruption, pH imbalance, and sustained inflammation.

The strongest predictors of cutaneous sensitization are inflamed skin and a high concentration of antigen(s), and biofilms increase the concentration of organisms per unit area (Fluhr et al., 2008). All our dermatitis subjects had inflamed skin but only those with a history of childhood flexural dermatitis had a high abundance of *Alternaria* species. This may increase the risk of sensitization to *Alternaria* in the affected skin of patients with AD (Hernandez-Ramirez et al., 2021). Thus, increased concentrations of cutaneous *Staphylococcus* and *Alternaria* biofilm matrix may provide an opportunity for immune sensitization and subsequent *Alternaria*-triggered asthma (Saunders et al., 2012; Jinnestal et al., 2014). Such interactions in AD may exacerbate skin barrier defects and inflammatory lesions, and protect pathogens (in biofilms) from antimicrobial agents. Importantly, the mechanisms behind these interactions are not completely understood, and thus, further studies are warranted.

Our findings, taken in the context of prior literature, suggest that biofilms of *Staphylococcus* and *Alternaria* may bidirectionally worsen the skin barrier and promote allergic sensitization. A recent clinical trial has reported that the use of emollients did not show a significant impact in reducing the risk of AD in high-risk infants compared to controls (Chalmers et al., 2020). Furthermore, the use of emollients was associated with an increased risk of infection and increased incidence of food allergy in infants with increased frequency of application suggested to be caused through transcutaneous sensitization (Chalmers et al., 2020; Perkin et al., 2021). Therefore, emollients may not be beneficial for patients with dermatitis complicated by the presence of biofilms, and this has clinical implications for patient counseling. Biofilm is challenging to identify from a clinical point of view but maybe hypothesized from the fast recurrence of cutaneous infections and antibiotic lack of response. Thus, emollients may not be beneficial for patients with AD complicated by the presence of biofilms, although ceramide predominate moisturizers warrant further study (Elias, 2018).

Another potential treatment target in managing AD is to modulate the skin microbiome, first, to prevent the *Staphylococcus*/*Alternaria* interaction and, second, to increase the abundance of beneficial microbiota that has been reported to play a significant role in regulating skin barrier formation and repair *via* aryl hydrocarbon receptor in keratinocytes (Uberoi et al., 2021).

Going forward, a deeper understanding of the role of *Staph/Alternaria* biofilms in both dermatitis and downstream development of fungal-triggered asthma may lead to the development of interventions to disrupt these biofilms. Avoidance of occlusive emollients should be studied as a strategy to discourage biofilm formation. Patch testing for delayed type sensitization to *Staph* and *Alternaria* would also be useful to expedite treatment with a biological drug, as avoidance of *Staph* and *Alternaria* as contact allergens is not feasible. Currently, patch testing to an extended screening series with the addition of all contact allergens obtained from exposure history is needed to determine if avoidance therapy for contact dermatitis will suffice to cure dermatitis.

The generalizability of our findings is limited by a small sample size, which enhanced the interpersonal variability recorded in microbiome studies and is one of the primary reasons for non-significance in small sample size studies. Another caveat is the fact that all subjects were recruited from a referral-based specialty clinic introducing potential selection bias (patients whose dermatitis was more refractory to entry-level treatments and required referral to tertiary care). The small number of subjects prevented us from addressing the role of potential confounding factors including gender, skin sampling location, race, age, and use of topical corticosteroids or prior response to systemic or topical antibiotic treatments. Furthermore, although sequencing-based analysis is a more sensitive detector of *S. aureus* than culturing techniques, we cannot provide a threshold at which the relative abundance of *S. aureus* would translate to a positive clinical culture, which makes these data difficult to compare to prior studies performed using culturing techniques (Byrnes et al., 2010).

Despite these caveats, our study represents a novel resource in the investigation into the interactions between skin bacteriome and mycobiome in AD which may help to personalize therapy for the diverse cohort of patients with dermatitis.

## Materials and methods

### Ethical approval

This study was approved by the Institutional Review Board of University Hospitals Cleveland Medical Center (UHCMC) (IRB# # 05-95-03).

### Study design

Thirteen study participants (age ≥ 18 years) were recruited from the UHCMC outpatient dermatology clinic by a physician for signs of barrier disruption (Supplementary Figure 4). Patients were divided into two cohorts based on dermatologic history: patients with childhood-onset, flexural dermatitis constituted atopic dermatitis (AD) group, and patients with adult-onset dermatitis with relevant positive patch test (non-Atopic, allergic contact dermatitis control group). The use of non-atopic controls as the comparator group allowed us to control for the microbial changes associated with broken, inflamed skin and identify microbiome changes uniquely associated with genetic differences evident in childhood, thus providing a more stringent control than could a cohort of healthy individuals with intact epidermis.

Table 1 shows the subject demographics: the mean age was 52.5 years with a range of 31–68 years. There were four women and nine men. The subjects with a history of childhood flexural dermatitis were slightly younger (mean age 47.3 years), compared to 56.0 years for the subjects without a history of childhood flexural dermatitis.

**Table 1:** Study participant demographic data.

**Disease classification**	**Number of patients**	**Age**	**Gender**	**Respiratory atopy status**	**Affected swab sites**
AD	2	31	F	Eczema, asthma, rhinitis	RAF
		58		Eczema, asthma	RAF, N
CD	7	41	M	None	N, AV, APA
		53		Seasonal rhinitis	AF, VW
		55		Seasonal rhinitis	AF, AV, APA
		56		Seasonal rhinitis	RAF, VW,
		59		None	RAF, N
		66		Asthma	AF, APA
		68		None	RAF, N, AF, VW
AD+CD	4	34	F	Eczema, rhinitis	AF, VW,
		41		Childhood eczema	N, VW
		53	M	Eczema, asthma, rhinitis	N, AV
		67		Eczema, asthma	N

AD, atopic dermatitis patient group; AD + CD, atopic dermatitis with positive patch test patient group; CD, allergic contact dermatitis patient group; F, female; M, male; RAF, retro-auricular fold; N, neck; AF, antecubital fossa; VW, volar wrist; AV, axillary vault; APA, anterior/posterior axillary line.

Each patient was evaluated by physician inspection for visible barrier disruption in the six eligible sites for swabbing: neck, anterior or posterior axillary line, axillary vault, volar wrist, antecubital fossa, and retro-auricular fold. We collected swabs from four to six of these potential sites per patient, depending on that patient's disease distribution, with the goal of collecting an equal number of affected and unaffected swabs per patient. Prior to swabbing, we categorized each site as either affected, if there was evidence of severe barrier disruption indicated by vesicles, papules, excoriations, or lichenification, or unaffected if the skin barrier was visibly intact.

Samples were collected using dry, sterile, cotton-tipped swabs, and rolled gently over the surface of the area of interest. Skin swabs were placed in tubes containing 500 μl glass beads (Sigma-Aldrich) and 1 ml ASL™ lysis buffer (Qiagen DNA Extraction Kit). Samples were stored at −80°C until processing. Additional details of the study design are provided in Supplementary methods. A total of 29 swabs were collected from affected skin and 29 from unaffected skin.

### Microbiome analyses

Fungal and bacterial genomic DNA was isolated from the collected swabs using QiaAmp DNA Mini Kit (Qiagen) following the manufacturer's instructions, and their microbiome profile was determined using the Internally Transcribed Spacer 1 (ITS1) and 16S rDNA regions for fungi and bacteria, respectively, as described previously by our group (Mukherjee et al., 2014, 2017; Chandra et al., 2016). The Greengenes V13_8 database was used for the taxonomic identification of the 16S RNA and the UNITE (7.2) database was used for the taxonomic identification of the ITS. In brief, the ITS1 region was amplified using ITS1F (CTTGGTCATTTAGAGGAAGTAA) and ITS 2 (GCTGCGTTCTTCATCGATGC) primers, and the V4 region of the 16S rRNA gene was amplified using 16S-515F: GTGCCAGCMGCCGCGGTAA and 16s-806R: GGACTACHVGGGTWTCTAAT primers. PCR reactions were carried out on 100 ng template DNA, in a 50 μl (final volume) reaction mixture. PCR products were sheared for 20 min using Ion Shear Plus Fragment Library Kit (Life Technologies, NY, USA). Amplicon library was generated with sheared PCR products using Ion Plus Fragment Library kits (<350 bp) according to the manufacturer's instructions, barcoded with Ion Xpress™ Barcode Adapter, and ligated with adaptors.

### Sequencing, classification, and analysis

The adapted barcoded libraries were equalized using the Ion Library Equalizer kit to a final concentration of 100 pM, pooled and diluted to 26 pM, and attached to the surface of Ion Sphere particles (ISPs) using an Ion PGM Template OT2 200bp kit v2 (Life Technologies, USA) according to the manufacturer's instructions, *via* emulsion PCR. The quality of ISPs' templates was checked using the Ion Sphere™ Quality Control Kit (Part no. 4468656) with the Qubit 2.0 device. Sequencing of the pooled libraries was carried out on the Ion Torrent Personal Genome Machine (PGM) system using the Ion Sequencing 200 kit v2 (all from Life Technologies) for 150 cycles (600 flows), with a 318-chip following the manufacturer's instructions. De-multiplexing and classification were performed using the Qiime 1.6 platform. The resulting sequence data were trimmed, reads with Phred score <Q25 and <100 bp were removed (Caporaso et al., 2010), and *de novo* operational taxonomic units (OTUs) were clustered using the Uclust algorithm (90% similarity threshold to represent the phyla, 94% similarity threshold to present the genus, and 97% sequence similarity representing the species) (Edgar, 2010). Classification at the species level was referenced using the UNITE 5.8s database for fungi and Greengenes V13_8 using the blastn function for bacteria. The sequences were assigned to the best BLAST hit with a minimum of 98.5% sequence homology (Altschul et al., 1990; Kõljalg et al., 2013). Principal coordinates analysis (PCoA), alpha/beta diversity, correlation analyses, and non-parametric analyses (since equal variance could not be assumed due to the small sample size) were conducted using the statistical programming language *R* and relevant packages (*vegan, PMCMR*, and *corrplot*) (Morgan and Huttenhower, 2012; Pohlert, 2014; Wei and Simko, 2016; Oksanen et al., 2017). Taxa with a frequency of at least 20% (and relative abundance >1%) were considered to comprise the core biome. *P* < 0.05 were considered significant.

### Biofilm assessment

Single- or mixed-species biofilms were formed and quantitated as described previously (Chandra et al., 2008). The optical density of biofilms was calculated as described above using the XTT assay for metabolic activity (additional details are provided in Supplementary methods).

### Real-time PCR assay

Primary keratinocytes were obtained from discarded neonatal foreskins (using an IRB-exempt protocol), grown to confluence, and exposed to 10% conditioned media obtained from cultured *Staphylococcus aureus* (SA) only, SA + Alternaria, Alternaria only, or media alone. Total RNA was extracted and analyzed for expression of targeted cytokines and regulatory genes using specific primers and the Superscript III reverse transcription kit (Life Technologies). Relative quantity (RQ) was plotted for each host variable and compared between the three groups using ANOVA and Tukey's *post-hoc* tests using GraphPad Prism (ver. 7.0). Additional details are provided in Supplementary methods.

### Bioinformatics and statistical analyses

The statistical programming language *R* and related packages were used for diversity and correlation analyses, and Kruskal–Wallis (non-parametric) analysis of variance using abundance data (Morgan and Huttenhower, 2012). Principal coordinates analysis (PCoA) was conducted with identified reads/OTUs using classical multidimensional scaling (functions *dist*/Euclidean, *cmdscale*) to analyze the distribution of dissimilarities and hierarchical clustering in dendrograms in the R package *vegan*. The homogeneity of groups and beta diversity were evaluated using *adonis* (which explores the differences in group means and implements a multivariate analysis of variances using distance matrices) and *betadisper* (which studies the differences in group homogeneities, similar to Levene's test of the equality of variances) functions in *vegan*. Diversity was analyzed using the Shannon diversity index (characterizes species diversity) and abundance-based richness estimates (observed, bias-corrected Chao, and ACE) were calculated using *vegan* (Oksanen et al., 2017). Taxa with a frequency of at least 20% (and relative abundance >1%) were considered to comprise the core biome in our study. Intra- and inter-kingdom correlations were determined using the *corrplot* package in *R*. Proportions of specific bacteria and fungi in tested samples were compared using non-parametric tests (PMCMR package). Due to the small sample size, equal variance across data could not be assumed and data were analyzed using non-parametric tests followed by Dunn's test (Bonferroni *post-hoc* correction), as applicable. Spearman Rank correlation analyses were conducted to identify inter- and intra-kingdom associations and microbial interactions with AD-related host variables. *P* < 0.05 were considered significant.

## Data availability statement

The original contributions presented in the study are included in the article/Supplementary material, further inquiries can be directed to the corresponding author/s.

## Ethics statement

The studies involving human participants were reviewed and approved by Institutional Review Board of University Hospitals Cleveland Medical Center IRB# 05-95-03. The patients/participants provided their written informed consent to participate in this study.

## Author contributions

Methodology and project conduction: MH. Conceptualization and project administration: PM. Writing—original draft: MH and AG. Writing—review and editing: MG, TM, SN, and GD. All authors contributed to the article and approved the submitted version.

## Funding

Funding support is acknowledged from the National Eczema Association and Steris Foundation (to PM) and from the NIH (R01DE024228 to MG and PM), and the NIH-funded Skin Diseases Research Center (NIAMS P30 AR039750). Funders had no role in in study design; the collection, analysis and interpretation of data, in the writing of the report, or in the decision to submit the article for publication.

## Conflict of interest

The authors declare that the research was conducted in the absence of any commercial or financial relationships that could be construed as a potential conflict of interest.

## Publisher's note

All claims expressed in this article are solely those of the authors and do not necessarily represent those of their affiliated organizations, or those of the publisher, the editors and the reviewers. Any product that may be evaluated in this article, or claim that may be made by its manufacturer, is not guaranteed or endorsed by the publisher.

## Supplementary material

Supplementary Figure 1 Richness and diversity (Shannon index) of bacterial and fungal taxa in atopic and non-atopic patients. Diversity was analyzed using the Shannon diversity index (characterizes species diversity) and abundance-based richness estimates (observed, bias-corrected Chao, and ACE) were calculated using *vegan*. **(A–D)** Richness, **(E–H)** Shannon diversity index.

Supplementary Figure 2 The proportion of selected fungal genera in atopic and non-atopic skin.

Supplementary Figure 3 Effect of *Staphylococcus* on *Alternaria* biofilms. Scanning electron microscopy images of **(A)** *Alternaria* (AA), mag × 3,500, **(B)** *Staphylococcus* (SA), mag × 3,500, **(C)** *Alternaria* + *Staphylococcus* (AA + SA), mag × 3,500, **(D)** *Alternaria* + *Staphylococcus* (AA + SA), mag × 10,000. **(E)** Biofilm formation of AA (measured by absorbance of metabolic activity) in presence of increasing densities of SA cells. EPS, extracellular polysaccharide matrix.

Supplementary Figure 4 Graphic illustration of the study design.
