# Supplementary figures and images for: Cutaneous dysbiosis may amplify barrier dysfunction in patients with atopic dermatitis
Source: Front Microbiol. 2022 Nov 14;13:944365. doi: 10.3389/fmicb.2022.944365 (PMC9701744; doi:10.3389/fmicb.2022.944365)

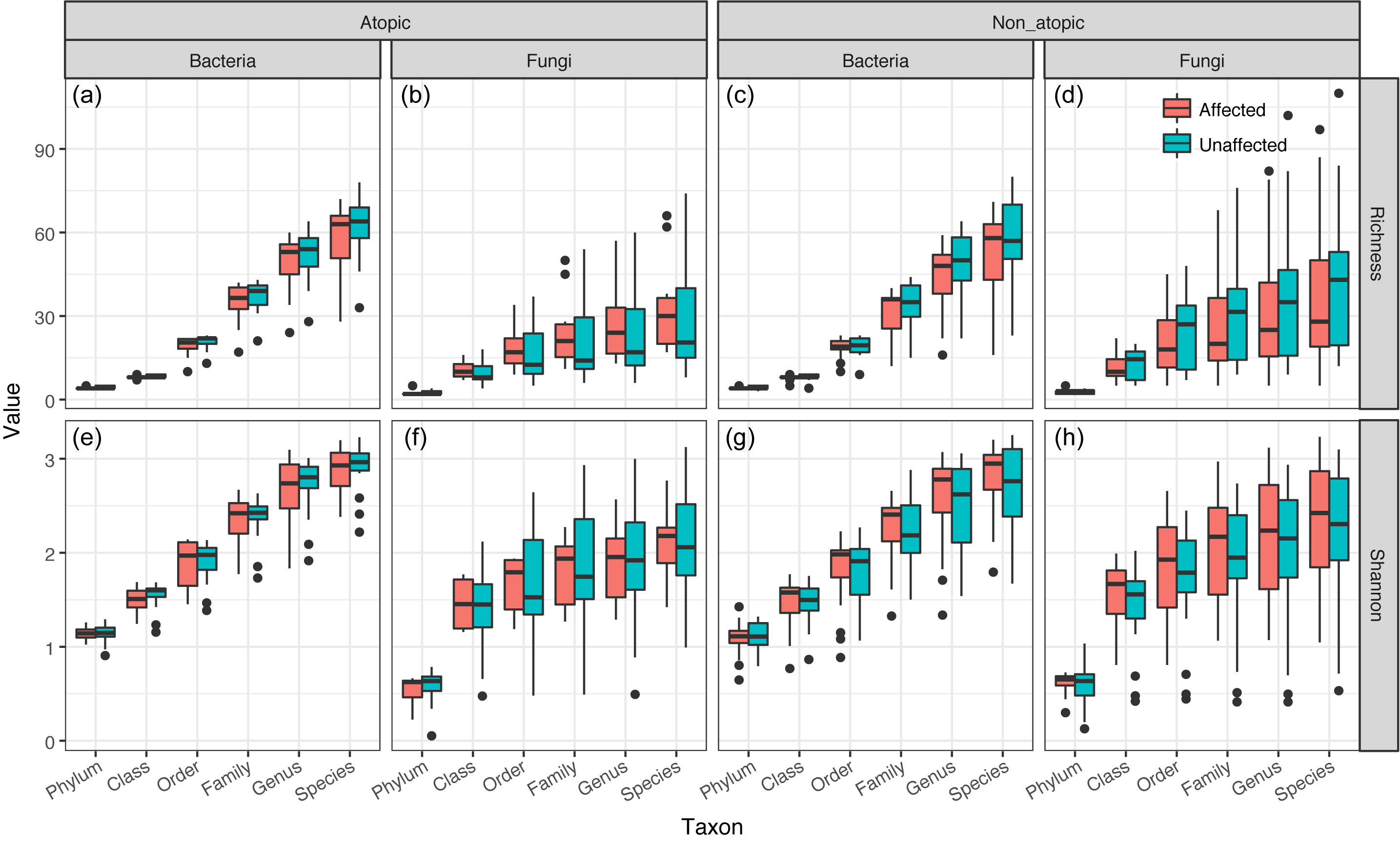

Supplement: Supplementary Figure 1 — Richness and diversity (Shannon index) of bacterial and fungal taxa in atopic and non-atopic patients. Diversity was analyzed using the Shannon diversity index (characterizes species diversity) and abundance-based richness estimates (observed, bias-corrected Chao, and ACE) were calculated using vegan. (A–D) Richness, (E–H) Shannon diversity index. [file Image_1.TIF]

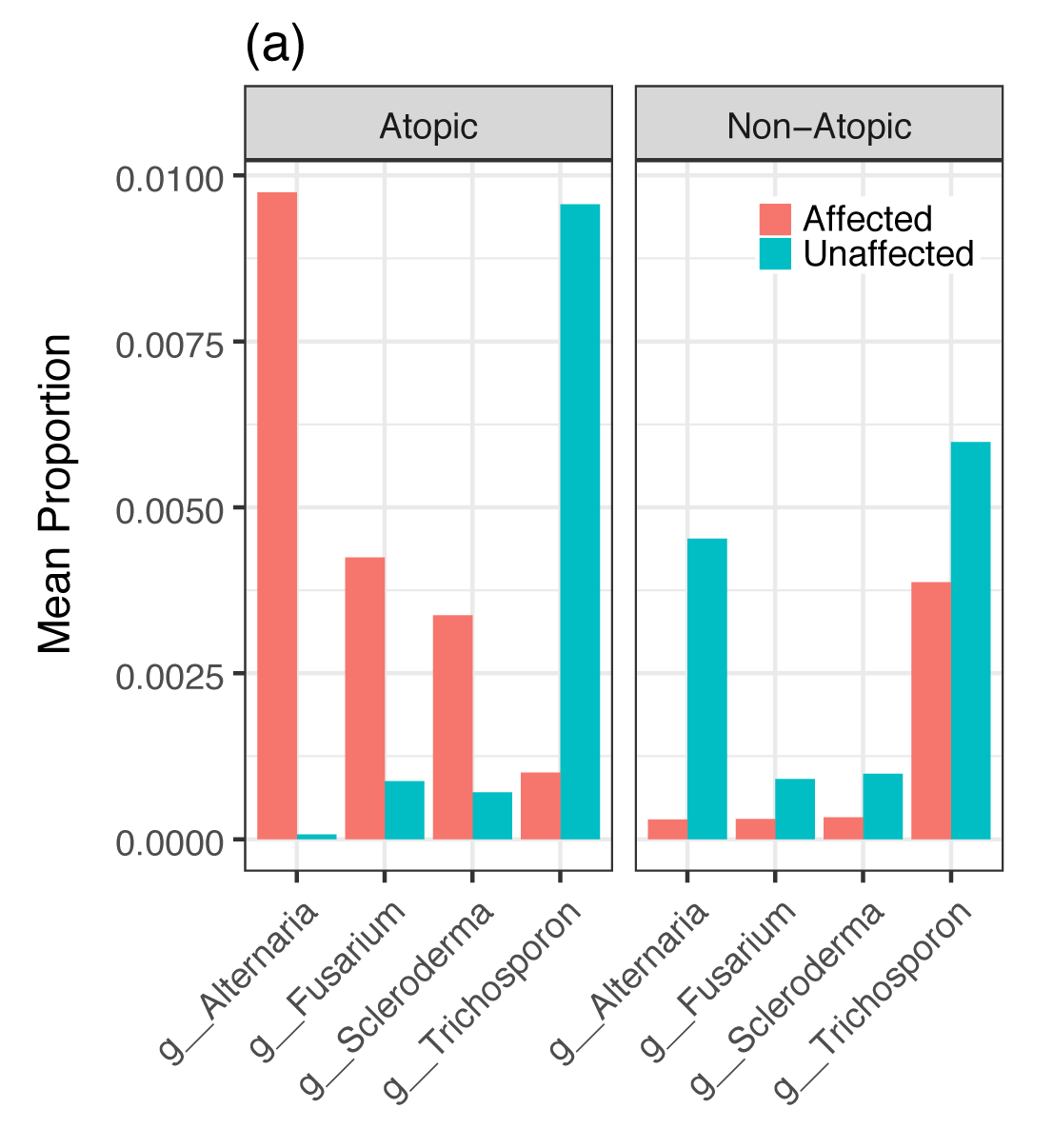

Supplement: Supplementary Figure 2 — The proportion of selected fungal genera in atopic and non-atopic skin. [file Image_2.TIF]

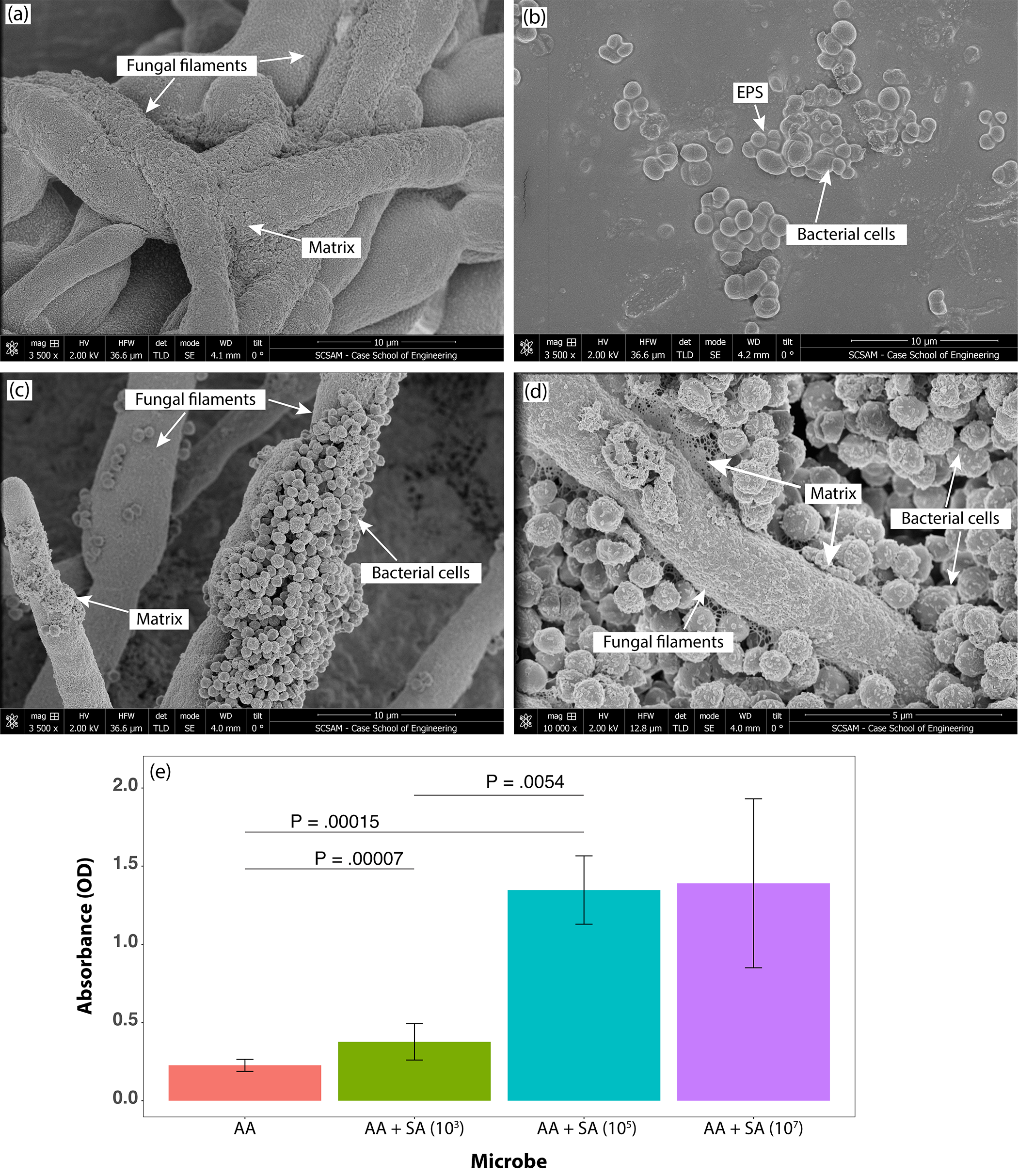

Supplement: Supplementary Figure 3 — Effect of Staphylococcus on Alternaria biofilms. Scanning electron microscopy images of (A) Alternaria (AA), mag × 3,500, (B) Staphylococcus (SA), mag × 3,500, (C) Alternaria + Staphylococcus (AA + SA), mag × 3,500, (D) Alternaria + Staphylococcus (AA + SA), mag × 10,000. (E) Biofilm formation of AA (measured by absorbance of metabolic activity) in presence of increasing densities of SA cells. EPS, extracellular polysaccharide matrix. [file Image_3.TIF]

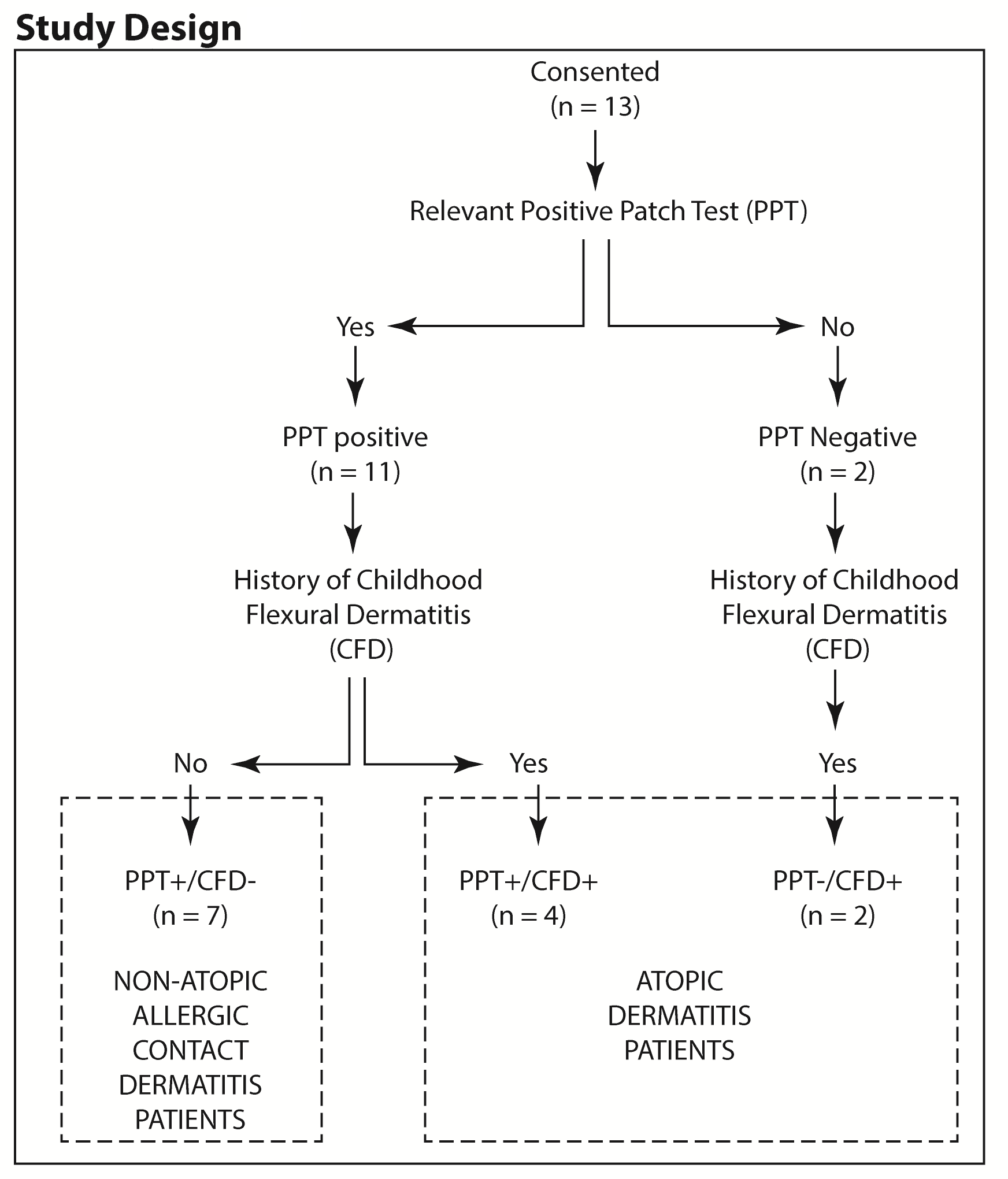

Supplement: Supplementary Figure 4 — Graphic illustration of the study design. [file Image_4.TIF]
