# Supplementary material for: Cutaneous dysbiosis may amplify barrier dysfunction in patients with atopic dermatitis
Source: Front Microbiol. 2022 Nov 14;13:944365. doi: 10.3389/fmicb.2022.944365 (PMC9701744; doi:10.3389/fmicb.2022.944365)
Supplement: Supplementary file 7 [file Table_3.DOCX]

**SUPPLEMENTARY MATERIALS AND METHODS**

**DNA Extraction:**

Fungal and bacterial genomic DNA was isolated and purified with the QiaAmp DNA Mini Kit (Qiagen) following the manufacturer’s instructions with minor modifications. Briefly, 3 additional bead-beating steps (Sigma-Aldrich beads, diameter = 500 µm) with the MP Fastprep-24 (speed setting of 6, 3 runs of 60s) were performed. The quality and purity of the isolated genomic DNA was confirmed spectrophotometrically using NanoDrop 2000 device (Fisher Scientific SAS, Illkirch, France). DNA concentration was quantified using the Qubit 2.0 instrument applying the Qubit dsDNA HS Assay (Life Technologies, USA). Extracted DNA samples were stored at -20°C.

**Biofilm Formation:**

Single- or mixed-species biofilms were formed and quantitated as described previously.[72] For single-species biofilms, *Alternaria alternata* (AA) strain ATCC 96153 was plated on Sabouraud dextrose (SD) agar and incubated at 30 degrees for 7 days. After this incubation, the plates were swabbed completely with a cotton swab which was placed into 10mL of phosphate-buffered saline (PBS) and diluted to 100x for counting. The appropriate volume of PBS was diluted in SD broth to produce a final concentration of 1x10^7^ cells/mL. 100 uL of the final dilution of cells were then placed in each well of a flat-bottomed, 96 well plate and 100 uL of SD broth was placed in the negative control wells. Plates were then incubated overnight at 30 degrees C to promote biofilm formation. Following biofilm formation, the media was carefully aspirated from each well and the plates were washed three times with sterile PBS, careful to prevent excessive disruption of the biofilm. After the final wash, the plate was drained completely and inspected for visible biofilms. Biofilms were also assessed with fluorescent microscopy at this stage.

For mixed-species biofilms, *Alternaria alternata* MRL 23378, a clinical isolate, was incubated on SD agar and prepared as above. The appropriate dilution of spores was made in a 1:1 SD/brain heart infusion (BHI) mixed broth. *S. aureus* (SA) strain ATCC 43300 was streaked onto a BHI agar plate, incubated for 24 hours, at 37 degrees C, and then diluted using a 0.5 McFarland turbidity standard at 625 nm. Bacterial cells were then serially diluted to 2x10^3^, 2x10^5^, and 2x10^7^ cells/mL in the same 1:1 SD/BHI mixed broth. 50 µL of the AA dilution was added to each well. 50 µL of SA cells were added to each well by row, increasing concentration with each row, and 50 µL of mixed broth was added to control wells. The plate was then incubated for 48 hours at 30 degrees C and washed as above. Biofilms were again assessed with fluorescent microscopy.

Biofilms were quantified via colorimetric, metabolic activity assays using 2, 3-bis (2-methoxy-4-nitro-5-sulfophenyl)-5-((phenyl amino) carbonyl)-2H-tetrazolium hydroxide (XTT). 100 μL of XTT/menadione solution was added to each well including the negative control well followed by incubation in the dark for 3 hours. Following incubation, 80 μL of the resulting colored supernatant was removed from each well and transferred to the corresponding wells of a second plate, which was then read in a MTP reader at 490 nm.

**Scanning Electron Microscopy:**

Biofilms were formed on catheter discs by incubating discs in the presence of AA MRL 23378, SA ATCC 43300, or a co-culture of AA and SA. Pre-treated catheter discs were allowed to adhere for 90 minutes with inoculum of AA, SA, or both, respectively. Discs were then transferred to new wells containing appropriate growth media and biofilms were allowed to mature for 48 hours. Mature biofilms were fixed with 2% glutaraldehyde followed by washes with sodium cacodylate buffer, 1% osmium tetraoxide, 1% uranyl acetate and dehydration through a series of ethanol solutions, from 25% (vol/vol) to absolute ethanol. Prepared samples were then sputter coated with Au/Pd (60/40) and viewed with ESEM scanning electron microscope.

**Real-Time PCR Assay:**

Primary keratinocytes were obtained from discarded neonatal foreskins (using an IRB-exempt protocol), grown to confluence, and exposed to 10% conditioned media obtained from cultured SA only, SA + Alternaria, Alternaria only, or media alone. Keratinocyte were incubated for 16-18 h at 37 °C, and total RNA was extracted the cells using an RNeasy mini kit (Qiagen). RNA was reverse transcribed using the Superscript III reverse transcription kit (Life Technologies) according to the manufacturer's instructions. cDNA was amplified in the presence of specific primers and probes for GATA3, CLDN1, TLR2, TLR4, TSLP, FLG, IL-1α, IL-23, TNFα, IL-18, and 18S rRNA and TaqMan Universal Master Mix in a 96-well microtiter plate format on a StepOnePlus real-time PCR system (all from Applied Biosystems). Each PCR was performed in triplicate, using the following conditions: 2 min at 50 °C and 10 min at 95 °C, followed by 40 cycles of 15 s at 95 °C and 1 min at 60 °C. Fold change was determined using the ΔΔ^CT^ method using 18S as the endogenous control. Relative quantity (RQ) was plotted for each host variable and compared between the three groups using ANOVA and Tukey’ post-hoc tests using GraphPad Prism (ver. 7.0).
